# Targeting IL-17/NF-κB/VAChT/Rho-kinase signaling and oxidative stress in exacerbated chronic allergic inflammation: functional and therapeutic implications of IL-17 blockade

**DOI:** 10.1590/1414-431X2026e15063

**Published:** 2026-03-30

**Authors:** L.N. Camargo, T.M. dos Santos, B.M. Saraiva-Romanholo, E.A. Leick, C.M. Prado, R.F. Righetti, I.F.L.C. Tibério

**Affiliations:** 1 Departamento de Clínica Médica, Faculdade de Medicina, Universidade de São Paulo, São Paulo, SP, Brasil; 2 Serviço de Reabilitação, Hospital Sírio-Libanês, São Paulo, SP, Brasil; 3 Departamento de Biociências, Universidade Federal de São Paulo, Santos, SP, Brasil

**Keywords:** Inflammation, Lipopolysaccharide, Anti-IL-17, Lung remodeling, Oxidative stress, Exacerbation

## Abstract

Th17 cytokines play a central role in the pathophysiology of chronic allergic pulmonary inflammation, influencing multiple signaling pathways that promote inflammation, oxidative stress, and airway remodeling. We evaluated the modulation of the NF-κB, VAChT, and Rho-kinase signaling pathways, and the effects of anti-interleukin (IL)-17 treatment on airway alterations in a murine model of chronic allergic inflammation were exacerbated by lipopolysaccharide (LPS). We studied airway hyperresponsiveness, inflammation, oxidative stress pathways, tissue remodeling, and the expression of various markers in male BALB/c mice with ovalbumin (OVA)-induced chronic allergic inflammation, with or without anti-IL-17 treatment. Twenty-four hours before the end of the experiment, the OVA-sensitized animals were treated with LPS (OVA-LPS-anti-IL-17). Mice treated with OVA-LPS-anti-IL-17 exhibited decreased elastance of the respiratory system after methacholine challenge, along with reduced infiltration of eosinophils, neutrophils, lymphocytes, and macrophages. Anti-IL-17 treatment also reduced the expression of TNF-α, TARC/eotaxin, IL-2, IL-4, IL-5, IL-6, IL-10, IL-13, IL-17, MMP-9, MMP-12, TIMP-1, TGF-β, iNOS, NF-κB, ROCK1, ROCK2, types I and III collagen, decorin, lumican, biglycan, fibronectin, and 8-iso-PGF2α in airway cells, as well as the mRNA expression of IL-17, VAChT, and arginase 1 in lung tissue, compared to the OVA and OVA-LPS groups (P<0.05), except for TNF-α and actin, which were not reduced compared to the OVA group, and Rrs, actin, and VAChT, which were not reduced compared to the OVA-LPS group. Thus, IL-17 blockade helped control bronchial hyperresponsiveness, modulate the IL-17/NF-κB/VAChT/Rho-kinase pathway, suppress chemokine expression, mitigate airway remodeling, and reduce NO-arginase expression in this asthma mouse model with LPS-induced exacerbation.

## Introduction

Although asthma is considered an allergic disease characterized by a classic Th2 inflammatory response, specific cytokines such as interleukin (IL)-17 are being studied to better understand the pathophysiology of asthma (1). IL-17 is associated with neutrophil recruitment and induces eosinophilia by inducing chemokine and growth factor secretion and airway remodeling (2). IL-17A may contribute to hyper-responsiveness by directly promoting the contractile responses of airway smooth muscle cells via nuclear factor-kappa B (NF-κB) activation and RhoA and Rho-associated protein kinase (ROCK) 2 induction (3)_._ Increased IL-17 expression was correlated with recruitment of eosinophils and neutrophils in an asthma model (4).

Airway eosinophilia in patients with severe asthma correlates with increased CC chemokine (CCL) expression. CCL17/TARC is a chemokine produced by monocytes primarily stimulated by Th2 cytokines or dendritic cells and modulates allergic inflammatory processes (5). In patients with asthma, airway epithelial cells produce high CCL11/eotaxin levels, which causes eosinophil migration and epithelial injury (6).

Several studies have highlighted the role of oxidative stress in the pathophysiology of asthma and have identified IL-17 as a key mediator associated with oxidative imbalance in this condition (7). The increased expression of inducible nitric oxide synthase (iNOS) is directly influenced by the proinflammatory action of IL-17 (8).

As these mediators can modulate airway inflammation in patients with asthma, inducing arginase activity can exacerbate nitric oxide (NO) deficiency and inflammation (9). The expression of types 1 and 2 arginase is regulated by a range of stimuli, including Th2 cytokines (such as IL-4, IL-10, and IL-13), NF-κB, and isoprostane PGF2α (8-iso-PGF2α) (10). We previously demonstrated that distal parenchymal constriction is related to increased arginase content and the number of iNOS-positive cells (11).

8-iso-PGF2α may function as a potent smooth muscle constrictor, and its association with tyrosine kinases Rho and ROCK may induce the activity of myosin light chain phosphatase (12). We previously reported that ROCK inhibition suppresses the oxidative stress pathway and attenuates maximal mechanical responses after an antigen challenge. Its combination with anti-IL-17 treatment potentiated these responses (13).

The proinflammatory effects of acetylcholine (ACh) are reportedly critical in mediating cell chemotaxis and inhibiting cytokine production, which helps counteract a continuous state of inflammation (14). ACh storage in synaptic vesicles is strongly related to the vesicular acetylcholine transporter (VAChT) activity. VAChT plays an essential role in maintaining pulmonary homeostasis and regulating key inflammatory signaling pathways such as NF-κB and Janus kinase (JAK)-signal transducer and activator of transcription (STAT) in lung cells (15).

Experimental models of chronic allergic asthma with lipopolysaccharide (LPS)-induced exacerbation, with or without anti-IL-17 treatment, demonstrated increased collagen deposition, the presence of proteoglycans in the airways and pulmonary parenchyma, and modulation of disease markers via IL-17 expression (16). Notably, Camargo et al. (16) reported significant effects of an IL-17 inhibitor evaluated specifically in the lung parenchyma. Building upon these findings and considering the critical role of airway cells in asthma pathophysiology, we assessed alterations in respiratory system resistance and elastance, the expression of multiple inflammatory markers, extracellular matrix remodeling, and oxidative stress in a model of LPS-induced chronic allergic inflammation.

## Material and Methods

### Animals and experimental model

The study protocol was approved by the Research Ethics Committee of Hospital das Clínicas of the Faculty of Medicine, University of São Paulo (Case No. 109/13). The experiment was conducted in two independent replicates using a total of 96 male BALB/c mice (n=8 per group; 6 groups per replicate), obtained from the University of São Paulo School of Medicine Laboratory Animal Center, which were handled in accordance with the Guidelines for the Care and Use of Laboratory Animals published by the Brazilian National Council for the Control of Animal Experimentation (CONCEA) (17). The average body weight of the animals was 20-25 g at the beginning of the sensitization protocol.

Mice were divided into six groups (n=8 in each group) based on the treatment administration: SAL: sterile saline solution via inhalation; OVA: ovalbumin solution via inhalation; SAL-anti-IL-17: sterile saline solution via inhalation and treated with an anti-IL-17 monoclonal antibody; OVA-anti-IL-17: ovalbumin solution via inhalation and treated with an anti-IL-17 monoclonal antibody; OVA-LPS: ovalbumin solution via inhalation and LPS via instillation; OVA-LPS-anti-IL-17: ovalbumin solution via inhalation and LPS via instillation and treated with an anti-IL-17 monoclonal antibody.

### Anti-IL-17 treatment

An anti-IL-17 (clone50104) neutralizing antibody (R&D Systems, UK) was administered intraperitoneally (*ip*; dose: 7.5 μg/application) 1 h before intratracheal instillation of LPS, following the protocol by Camargo et al. (16).

### Ovalbumin sensitization protocol

**Figure 1:**
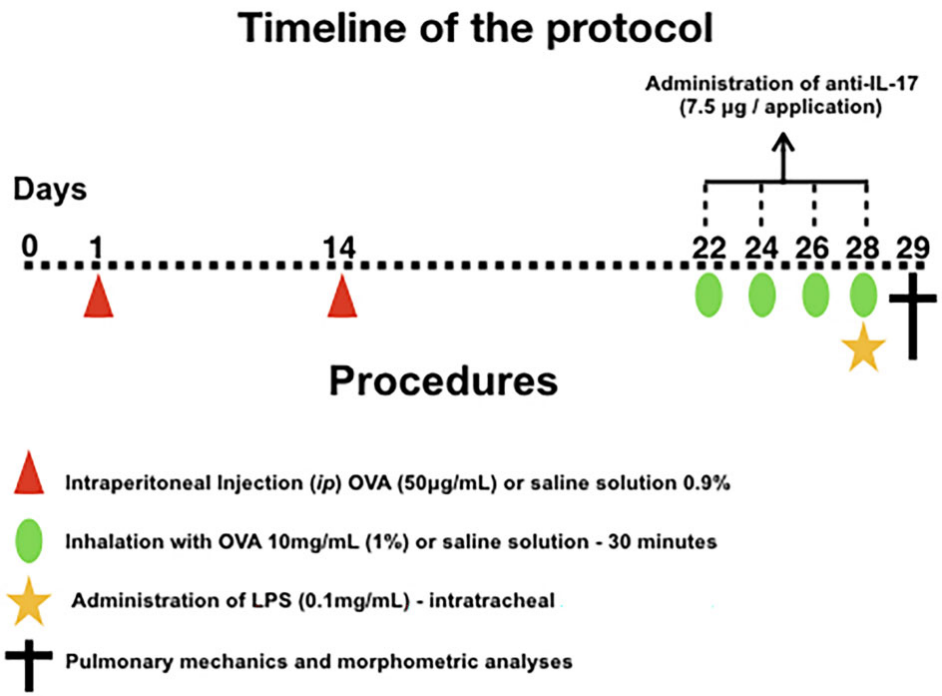
Timeline of the protocol for sensitization, induction of inflammatory response, and treatment. On days 1 and 14, mice from the OVA, OVA-LPS, OVA anti-IL-17, and OVA-LPS-anti-IL-17 groups were sensitized with OVA administered intraperitoneally (*ip*), whereas mice from the SAL control group received saline (*ip*). On days 22, 24, 26, and 28, 1 h before the inhalation challenge, mice in the treatment group were administered anti-IL-17 antibodies (*ip*), and 24 h after the experiment ended, mice from the OVA-LPS and OVA-LPS-anti-IL-17 groups underwent the intratracheal instillation of LPS (18). OVA: ovalbumin; LPS: lipopolysaccharide.

The sensitization protocol lasted 29 days. On days 1 and 14, BALB/c mice were administered a solution comprising 50 µg ovalbumin (Sigma-Aldrich, USA) and 6 mg Al(OH)_3_ adjuvant (Alumen, Pepsamar, Sanofi-Synthelabo SA, Brazil) in a total volume of 0.2 mL *ip* (Synthelabo SA). On days 22, 24, 26, and 28, the animals were subjected to the inhalation protocol for 30 min using an ultrasonic nebulizer (US-1000, ICEL, Brazil) coupled to an acrylic box (30×5×20 cm) diluted in 0.9% NaCl. Concurrently, the control group mice were administered (*ip*) saline solution (0.9% NaCl) and Al(OH)_3_ (6 mg) and were exposed to 0.9% saline aerosol for 30 min for the inhalation challenge. A schematic representation of the 29-day ovalbumin sensitization protocol used in this study is depicted in Figure 1.

### Murine model of ovalbumin sensitization with LPS-induced exacerbation

LPS treatment was performed by administering 20 μL phosphate-buffered saline (PBS) and 0.1 mg/mL *Escherichia coli* 0127: B8 (Sigma-Aldrich). LPS instillation was performed 24 h after the final antigen challenge on day 29, following the protocol described by Camargo et al. (16).

### Respiratory system mechanics

The animals were anesthetized with thiopental (50 mg/kg, *ip*) and tracheostomized on day 29. After tracheostomy, we attached a Harvard 683 ventilator (Harvard Apparatus, USA) and adjusted the following parameters: tidal volume of 10 mL/kg, respiratory rate of 120 cycles/min, and sinusoidal inspiratory flow curve. The animals received pancuronium (0.2 mg/kg *ip*) to abolish the ventilatory effort. Tracheal pressure (Ptr) was measured using a differential pressure transducer 142PC05D (Honeywell, USA), and the flow (V') was measured using a pneumotachograph (Fleisch-4.0, OEM Medical, USA); both instruments were connected to the tracheal cannula. An electronic system was used to integrate the flow circuit and accurately define the pulmonary volume changes (V). We recorded the tracings of Ptr, V', and V using a computer. These values were used to calculate the basal and maximal respiratory system resistance (Rrs) and elastance (Ers) after aerosol administration of methacholine (3, 30, and 300 mg/mL for 1 min). The following equation was applied: Ptr (t) = Ers × V (t) + Rrs × V' (t), where “t” denotes time (18).

### Bronchoalveolar lavage

After evaluating the respiratory mechanics of the mice, bronchoalveolar lavage was performed. Saline solution (0.5 mL) was instilled three times through the tracheostomy cannula with a syringe, and 1.5 mL of bronchoalveolar lavage fluid (BALF) was recovered. BALF was centrifuged at 790 *g* for 10 min at 5°C, with an average mean recovery of 80% (18). The cell pellet was resuspended in 300 μL saline using a vortex mixer. Subsequently, 100 μL of fluid was used to prepare slides for differential cell counting. The residual BALF was cytocentrifuged onto slides for 6 min at 450 *g* at 4°C and stained with Diff-Quick solution (Sigma-Aldrich [Merck], USA). Differential counts of neutrophils and eosinophils were performed using a light optical microscope at 1,000× magnification (18). Following the bronchoalveolar lavage, animals were euthanized by exsanguination of the abdominal aorta. Subsequently, their lungs were removed. One lung from each animal was used for morphometry, and the other for RT-PCR analysis.

### Airway histology and morphometry

**Figure 2:**
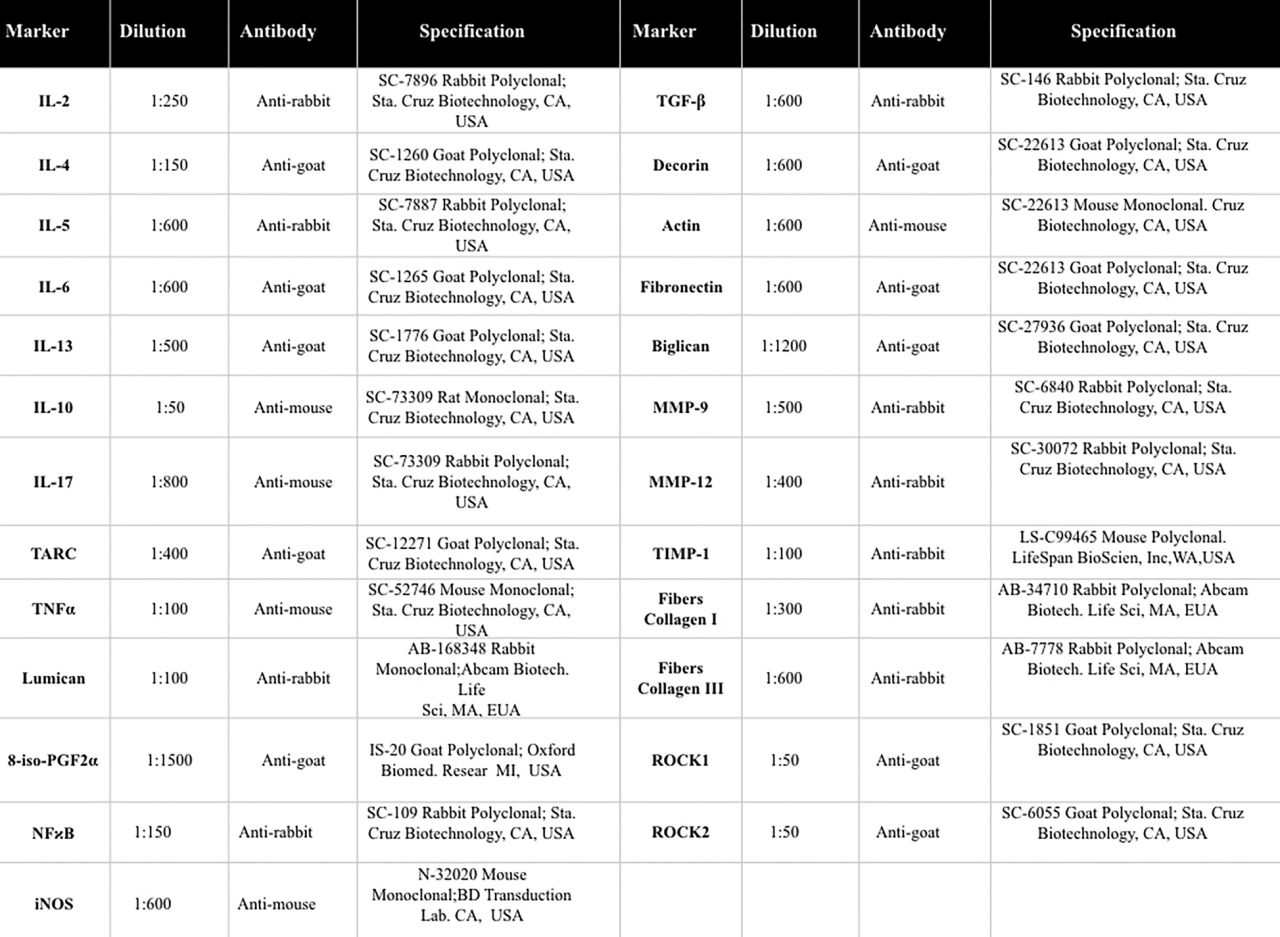
Description of the antibodies used in the immunohistochemical analysis.

The lungs were fixed with 4% formaldehyde at a constant pressure of 20 cmH_2_O for 24 h, maintained in 70% alcohol for up to 36 h, and prepared for histological processing. Pulmonary tissue fragments were then fixed and embedded in paraffin. Five-micrometer-thick slices were fixed on sheets and prepared with 3-aminopropyl-triethoxysilane silane (Sigma Chemical Co., USA). The slices were deparaffinized, rehydrated, treated with proteinase K (Sigma Chemical Co.) at 37°C for 20 min, incubated at room temperature for 20 min, and washed with 0.9% PBS. Endogenous peroxidases were blocked by incubating with 3% hydrogen peroxide (H_2_O_2_) at 10 V (3×10 min). This was followed by overnight incubation with the indicated antibodies (Figure 2). After 24 h, the slides were washed with PBS and incubated with secondary antibodies using the ABCKit with Vectastain (Vector Elite-PK-6105 anti-goat), PK-6101 (anti-rabbit), and PK-6102 (anti-mouse). To observe cells expressing different proteins, the slides were washed in PBS and stained with 3,3′-diaminobenzidine (DAB) chromogen (Sigma Chemical Co.). Slide sections were contrasted with Harris hematoxylin (Merck, Germany) and mounted using Entellan microscopy resin (Merck) (7).

For morphometric analysis, we quantified the density of cells positive for tumor necrosis factor (TNF)-α, interleukin (IL)-2, IL-4, IL-5, IL-6, IL-10, IL-13, IL-17, CCL17/TARC, iNOS, p65-NF-κB, TGF-β, MMP-9, MMP-12, TIMP-1, ROCK1, and ROCK2 in the airway walls. A grid of known area (50 lines and 100 points) coupled to a microscope (E200MV, Nikon Corporation, Japan) was used for the analysis (19 [Weibel, 1963]). The reticule was applied to four randomly selected fields of the airway wall in 3 to 5 tissue sections per animal. The number of points coinciding with positively stained cells was divided by the total number of points over the airway wall area. The grid covered a total area of 10^4^ μm^2^. All analyses were performed at 1000× magnification, and results are reported as the number of positive cells per 10^4^ μm^2^. All quantifications were performed under blinded conditions (14).

Optical density analysis was used to determine the content of collagen fiber types I and III, actin, decorin, lumican, biglycan, fibronectin, and 8-iso-PGF2α. Images were captured using an image analysis system composed of a Zeiss Axioplan microscope (Germany) coupled with a video camera and transferred to a computer using an image scanner. Images were acquired and processed using Optimas v. 4.10 (Media Cybernetics, USA). For quantification, a polarizer was coupled to a microscope. For each animal, five airways were evaluated at a magnification of 1000×, and areas positive for types I, III, and V collagen fibers, actin, decorin, lumican, biglycan, fibronectin, and 8-iso-PGF2α are reported as a percentage of the total area of the airway wall. The slides were coded, and the researcher performed the analysis under blinded conditions (19).

### RNA extraction and reverse transcription-quantitative PCR (RT-qPCR)

One lung was removed from each mouse at the end of the mechanical evaluation. Total RNA was extracted from lung tissues using TRIzol (Invitrogen Life Technologies, USA), and the ratio of absorption at 260/280 nm and 260/230 nm was determined. Total RNA was reverse-transcribed to cDNA using the SuperScript III kit (Invitrogen Life Technologies). Gene expression was assessed by RT-qPCR using a rotor gene (Qiagen, Netherlands) and SYBR Green as a fluorescent dye (Qiagen), with *GAPDH* as the control. The reaction conditions were as follows: 95°C for 5 min, followed by 40 cycles of 95°C for 5 s, and 60°C for 10 s. The primers used and annealing temperatures were as follows: arginase 1 (ARG-1): sense GCACTCATGGAAGTACACGAGGAC-(5′-3′), antisense CCAACCCAGTGATCTTGACTGA-(5′-3′); VAChT: sense CCCTTTTGATGGCTGTG-(5′-3′), antisense GGGCTAGGGTACTCATTAGA; IL-1: sense TGAAGGTCAACCTCAAAGTCT-(5′-3′), antisense GAGGGATATCTAGGGTCTTCA-(5′-3′) (60°C; NM_10167164). Data were obtained as Ct values (Ct = cycle number in which the logarithmic PCR plots crossed a calculated threshold) and used to determine the ΔCt values. Data are reported as arbitrary units (AU) using the following transformation equation: expression = 1000 × (2^-ΔCt^) AU; (ΔCt = (Ct of the target gene) (18).

### Data analysis

Statistical analyses were performed using SigmaStat version 10 (Jandel Scientific, USA). Data are reported as means±SE. Differences among groups were assessed using one-way ANOVA and the Student-Newman-Keuls *t*-test as a *post hoc* test. Differences were considered statistically significant at P<0.05.

## Results

### Maximal responses to methacholine in mice from different treatment groups

**Figure 3:**
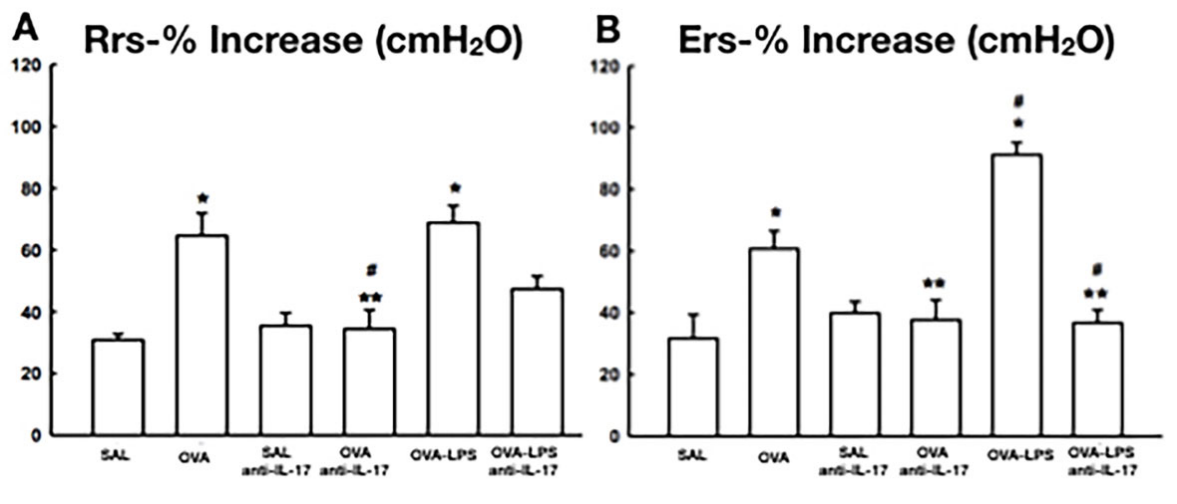
Effects of anti-interleukin (IL)-17 treatment on lung hyperresponsiveness, measured as percentage increase from baseline values of resistance (Rrs; **A**) and elastance (Ers; **B**) after methacholine challenge in all experimental groups. Data are reported as means±SE. *P<0.05 compared with SAL and SAL-anti IL-17 groups; ^#^P<0.05 compared with OVA group; **P<0.05 compared with OVA-LPS group; ANOVA. SE: standard error; SAL: saline; OVA: ovalbumin; LPS: lipopolysaccharide.

The Rrs and Ers percentages in the OVA and OVA-LPS groups were higher than in the control group (P<0.05). The percentages of Rrs and Ers in the OVA-anti-IL-17 group were lower than in the OVA and OVA-LPS groups (P<0.05). The %Ers in the OVA-LPS group was higher than in the OVA group. In the OVA-LPS-anti-IL-17 group, the %Ers was attenuated compared to the OVA-LPS and OVA groups (P<0.05), except for the %Rrs in the OVA-LPS-anti-IL-17 group, which showed no difference compared to the OVA-LPS group (Figure 3).

### Effects of anti-IL-17 treatment on VAChT expression

**Figure 4:**
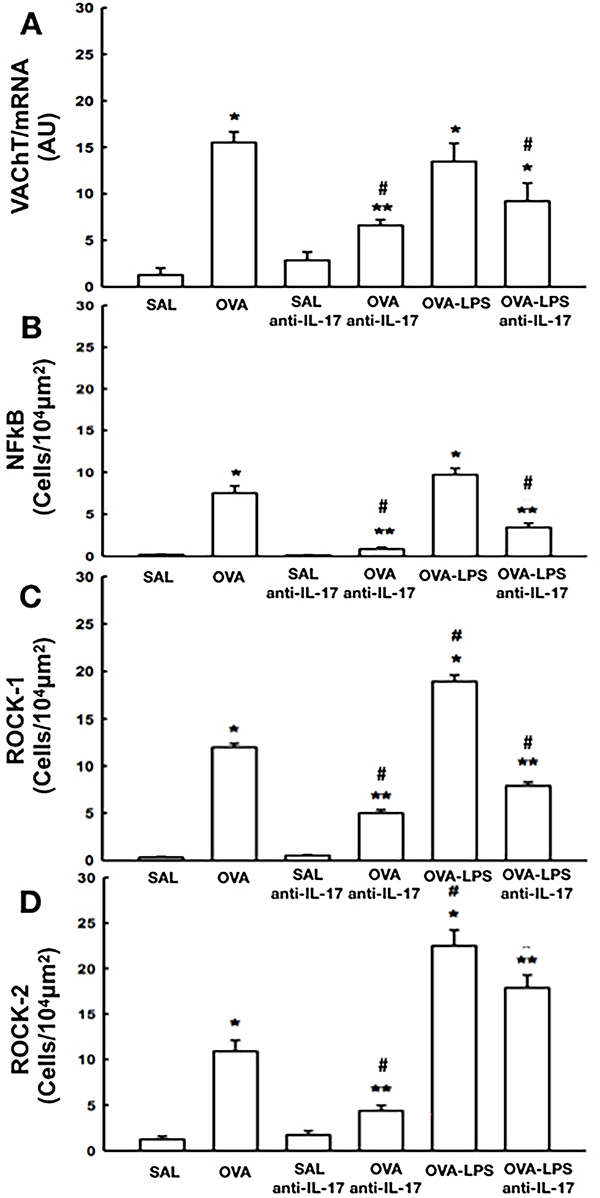
Effects of anti-interleukin (IL)-17 treatment on signaling pathways. Gene VAChT/mRNA expression (**A**); cells positive for p65-NFκB (**B**), ROCK1 (**C**), and ROCK2 (**D**) in all experimental groups. Data are reported as means±SE. *P<0.05 compared with SAL and SAL-anti IL-17 groups; ^#^P<0.05 compared with OVA group; **P<0.05 compared with OVA-LPS group; ANOVA. SE: standard error; AU: arbitrary units; SAL: saline; OVA: ovalbumin; LPS: lipopolysaccharide.

The OVA and OVA-LPS groups showed higher VAChT expression than the SAL and anti-IL-17 control groups (P<0.05) (Figure 4A). VAChT expression was lower in the OVA-anti-IL-17 and OVA-LPS-anti-IL-17 groups than in the OVA and OVA-LPS groups (P<0.05).

### Effects of anti-IL-17 treatment on cellular p65-NF-κB expression

The number of p65-NF-κB-positive cells in the OVA and OVA-LPS groups was higher than in the SAL and anti-IL-17 control groups (P<0.05) (Figure 4B). The number of p65-NF-κB-positive cells in the OVA-anti-IL-17 and OVA-LPS-anti-IL-17 groups was lower than in the OVA and OVA-LPS groups (P<0.05).

### Effects of anti-IL-17 treatment on ROCK1 and ROCK2 expression

The number of ROCK1- and ROCK2-positive cells was higher in mice from the OVA and OVA-LPS groups than in the control groups (SAL and SAL-anti-IL-17) (P<0.05) (Figure 4C and D). The number of ROCK1- and ROCK2-positive cells in mice from the OVA-anti-IL-17 group was lower than in the OVA and OVA-LPS groups (P<0.05). Anti-IL-17 treatment reduced the number of ROCK1-positive cells in mice from the OVA-LPS-anti-IL-17 group compared to the OVA and OVA-LPS groups (P<0.05); however, ROCK2-positive cells remained elevated compared to the OVA group.

### Evaluation of oxidative stress responses

The number of iNOS-positive cells, the volumetric fraction of 8-iso-PGF2α, and ARG-1 mRNA expression in the OVA and OVA-LPS groups were higher than in the SAL and anti-IL-17 control groups (P<0.05) (Table 1). These same parameters were lower in the OVA-anti-IL-17 and OVA-LPS-anti-IL-17 groups than in the OVA and OVA-LPS groups (P<0.05); however, in the OVA-LPS-anti-IL-17 group, ARG-1 mRNA expression was not attenuated compared to the OVA-LPS group (P<0.05).

**Table 1:** Oxidative stress markers in the airway and arginase-1 gene expression (absolute values).

Oxidative stress markers	SAL	OVA	SALanti-IL-17	OVAanti-IL-17	OVA-LPS	OVA-LPSanti-IL-17
iNOS (cells/10^4^ μm^2^)	1.58±0.26	6.20±1.00*	2.37±0.48	3.05±0.28^#^**	8.76±0.77*^#^	5.26±0.49**
8-iso-PGF2α (%)	6.83±0.90	15.28±1.15*	7.39±0.89	9.87±1.07^#^**	20.59±2.10*^#^	9.47±0.90^#^**
Arginase 1/mRNA (AU)	0.88±0.39	3.62±0.44*	1.24±0.48	1.65±0.30^#^**	7.64±1.29*^#^	2.95±1.52**

Morphometric evaluation was performed in the airways, and mRNA expression was analyzed in total lung tissue. Data are reported as means±SE. *P<0.05 compared with SAL and SAL-anti IL-17 groups; ^#^P<0.05 compared with OVA group **P<0.05 compared with OVA-LPS group; ANOVA. IL: interleukin; SAL: saline; OVA: ovalbumin; LPS: lipopolysaccharide.

### Effects of anti-IL-17 treatment on bronchoalveolar lavage fluid

The total number of cells and the differential counts for eosinophils, lymphocytes, and macrophages in the BALF of mice in the OVA and OVA-LPS groups were higher than in the SAL group (P<0.05). However, neutrophil counts were significantly increased only in the OVA-LPS group compared to the SAL group, with no significant difference between the OVA and SAL groups (P<0.05) (Table 2). Anti-IL-17 treatment significantly reduced the total number of cells, as well as the counts of eosinophils, lymphocytes, and macrophages in the BALF of mice in both the OVA-anti-IL-17 and OVA-LPS-anti-IL-17 groups (P<0.05). A reduction in neutrophil counts was observed exclusively in the OVA-LPS-anti-IL-17 group when compared to the OVA-LPS group (P<0.05), with no significant change in the OVA-anti-IL-17 group.

**Table 2:** Bronchoalveolar lavage fluid (BALF) findings and inflammatory markers in the airway (absolute values).

BALF	SAL	OVA	SALanti-IL-17	OVAanti-IL-17	OVA-LPS	OVA-LPSanti-IL-17
Total cells (10^4^/cells/mL)	1.01±0.22	33.22±4.71*	0.40±0.09	5.08±0.56^#^**	39.14±4.31*^#^	6.65±0.93^#^**
Neutrophils (10^4^/cells/mL)	0.08±0.03	0.51±0.21	0.03±0.02	0.33±0.15**	3.98±0.77*	1.03±0.35**
Eosinophils (10^4^/cells/mL)	0.01±0.01	22.38±5.84*	0.00±0.00	4.11±1.32^#^**	21.01±6.48*	3.71±1.38^#^**
Lymphocytes (10^4^/cells/mL)	0.24±0.14	5.14±0.83*	0.05±0.02	0.28±0.07^#^**	7.05±0.99*	0.71±0.26^#^**
Macrophages (10^4^/cells/mL)	0.37±0.13	5.45±1.91*	0.06±0.01	1.16±0.70^#^**	5.17±0.82*	1.16±0.34^#^**
Inflammatory markers						
TNF-α (cells/10^4^ μm^2^)	2.05±0.33	5.75±0.73*	0.92±0.42	6.65±0.71	6.26±1.36*	3.79±0.80**
CCL17/TARC (cells/10^4^ μm^2^)	1.06±0.20	5.19±0.24*	0.83±0.16	2.31±0.28^#^**	9.26±0.37*^#^	4.73±0.35**
CCL11/eotaxin (cells/10^4^ μm^2^)	0.60±0.12	3.89±0.42*	0.47±0.16	0.27±0.07^#^**	1.29±0.33*	0.48±0.08^#^**
IL-2 (cells/10^4^ μm^2^)	1.17±0.18	5.00±0.27*	1.38±0.17	3.02±0.27^#^**	7.64±0.35*^#^	1.95±0.10^#^**
IL-4 (cells/10^4^ μm^2^)	0.50±0.13	4.37±0.25*	0.40±0.04	2.30±0.37^#^**	7.80±0.42*	4.85±0.70**
IL-5 (cells/10^4^ μm^2^)	1.03±0.12	4.41±0.32*	1.19±0.18	1.74±0.14^#^**	6.84±0.81*	3.35±0.29**
IL-6 (cells/10^4^ μm^2^)	0.63±0.11	5.79±0.34*	0.88±0.13	1.52±0.14^#^**	7.81±0.34*^#^	2.23±0.28^#^**
IL-10 (cells/10^4^ μm^2^)	0.67±0.14	5.50±0.44*	0.37±0.12	1.52±0.14^#^**	9.30±0.51*^#^	2.93±0.30^#^**
IL-13 (cells/10^4^ μm^2^)	1.29±0.21	4.94±0.55*	0.71±0.10	2.26±0.37^#^**	9.00±0.55*^#^	4.66±1.00**

Data are reported as means±SE. *P<0.05 compared with SAL and SAL-anti IL-17 groups; ^#^P<0.05 compared with OVA group **P<0.05 compared with OVA-LPS group; ANOVA. IL: interleukin; TNF: tumor necrosis factor; SAL: saline; OVA: ovalbumin; LPS: lipopolysaccharide.

### Airway inflammation

The number of cells expressing TNF-α, CCL17/TARC, CCL11/eotaxin, IL-2, IL-4, IL-5, IL-6, IL-10, and IL-13 in the OVA and OVA-LPS groups was higher than that in the SAL and anti-IL-17 control groups (P<0.05) (Table 2). Anti-IL-17 treatment (OVA-LPS-anti-IL-17 group) reduced the number of cells expressing CCL17/TARC, CCL11/eotaxin, IL-2, IL-4, IL-5, IL-6, IL-10, and IL-13 compared to the OVA and OVA-LPS groups (P<0.05). No significant changes in IL-4 and IL-13 levels were observed when comparing the OVA-LPS-anti-IL-17 and OVA groups.

### Morphometric and immunohistochemical staining for IL-17

**Figure 5:**
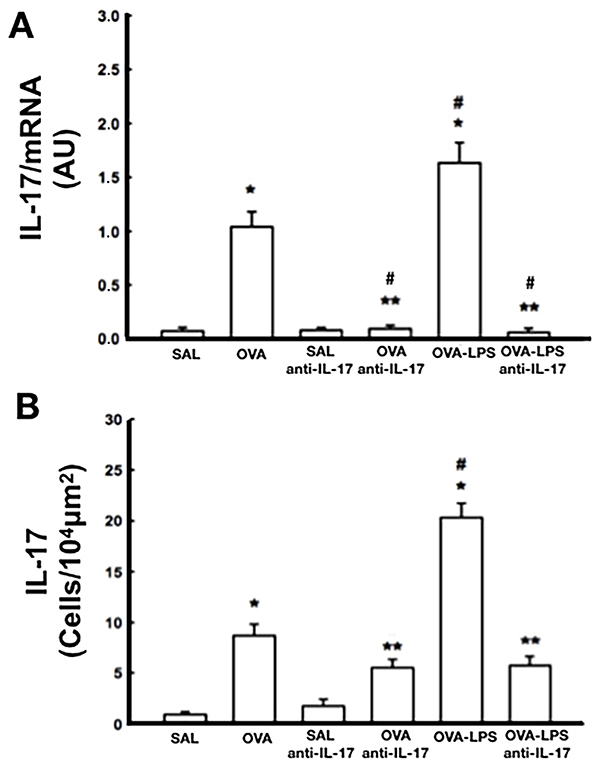
Interleukin (IL)-17 profile determined using RT-PCR (**A**) and morphometric analysis for IL-17-positive cells (**B**) in all experimental groups. Data are reported as means±SE. *P<0.05 compared with SAL and SAL-anti IL-17 groups; ^#^P<0.05 compared with OVA group; **P<0.05 compared with OVA-LPS group; ANOVA. RT-PCR: reverse transcription-polymerase chain reaction; SE: standard error; SAL: saline; OVA: ovalbumin; LPS: lipopolysaccharide.

The OVA and OVA-LPS mice showed higher IL-17 mRNA expression and a greater number of IL-17-positive cells than SAL and anti-IL-17 control mice (P<0.05) (Figure 5). IL-17 mRNA expression and the number of IL-17-positive cells were lower in OVA-LPS-anti-IL-17 mice than in OVA and OVA-LPS mice (P<0.05).

### Extracellular matrix remodeling

The volume fractions of type I and III collagen fibers, decorin, fibronectin, biglycan, lumican, and actin and the number of cells expressing TGF-β, MMP-9, MMP-12, and TIMP-1 were higher in the OVA and OVA-LPS groups than in the SAL and anti-IL-17 groups (P<0.05) (Table 3). Anti-IL-17 treatment (OVA-LPS-anti-IL-17 group) reduced the expression of these markers compared to the OVA and OVA-LPS groups (P<0.05). Changes in the expression of types I and III collagen fibers in response to anti-IL-17 treatment (OVA-LPS-anti-IL-17 group) were observed only in comparison with the OVA-LPS group (P<0.05).

**Table 3:** Extracellular matrix remodeling markers in the airway (absolute values).

Remodeling markers	SAL	OVA	SALanti-IL-17	OVAanti-IL-17	OVA-LPS	OVA-LPSanti-IL-17
Type I collagen fibers (%)	13.41±1.24	24.12±1.51*	12.63±0.88	19.04±2.06^#^	26.97±1.80*	22.97±0.65
Type III collagen fibers (%)	0.87±0.30	10.02±1.51*	1.04±0.35	1.13±0.20^#^**	15.01±1.47*^#^	10.25±1.14**
Decorin (%)	1.79±0.83	9.45±2.23*	1.64±0.47	5.61±0.90^#^**	16.90±0.84*^#^	4.23±0.76^#^**
Biglycan (%)	6.96±0.54	20.03±1.64*	7.52±0.40	10.00±0.84^#^**	19.00±1.22*	9.43±0.67^#^**
Lumican (%)	15.01±1.30	26.37±0.69*	16.23±0.45	14.78±0.01^#^**	24.24±1.09*	15.94±1.18^#^**
Fibronectin (%)	1.32±0.13	14.54±0.30*	1.30±0.09	11.42±0.79**	42.62±1.69*^#^	11.22±0.58**
Actin (%)	15.20±1.12	27.89±1.97*	16.26±1.96	16.17±1.91^#^**	25.40±3.06*	27.63±1.50
TGF-β (cells/10^4^ μm^2^)	1.00±0.35	22.04±1.88*	1.24±0.38	11.20±1.01^#^**	41.77±1.90*^#^	11.27±1.29^#^**
MMP-9 (cells/10^4^ μm^2^)	1.19±0.28	16.87±0.40*	1.73±0.19	5.82±0.54^#^**	23.70±2.40*^#^	10.81±1.07^#^**
MMP-12 (cells/10^4^ μm^2^)	0.40±0.08	13.31±0.80*	0.39±0.15	2.65±0.45^#^**	13.43±0.77*	3.06±0.51^#^**
TIMP-1 (cells/10^4^ μm^2^)	0.54±0.25	5.00±0.42*	0.62±0.12	1.68±0.26^#^**	8.99±1.40*^#^	3.79±0.65**

Data are reported as means±SE. *P<0.05 compared with SAL and SAL-anti-IL-17 groups; ^#^P<0.05 compared with OVA group. **P<0.05 compared with OVA-LPS group; ANOVA. TGF: transforming growth factor; SAL: saline; OVA: ovalbumin; LPS: lipopolysaccharide.

### Qualitative analysis

**Figure 6:**
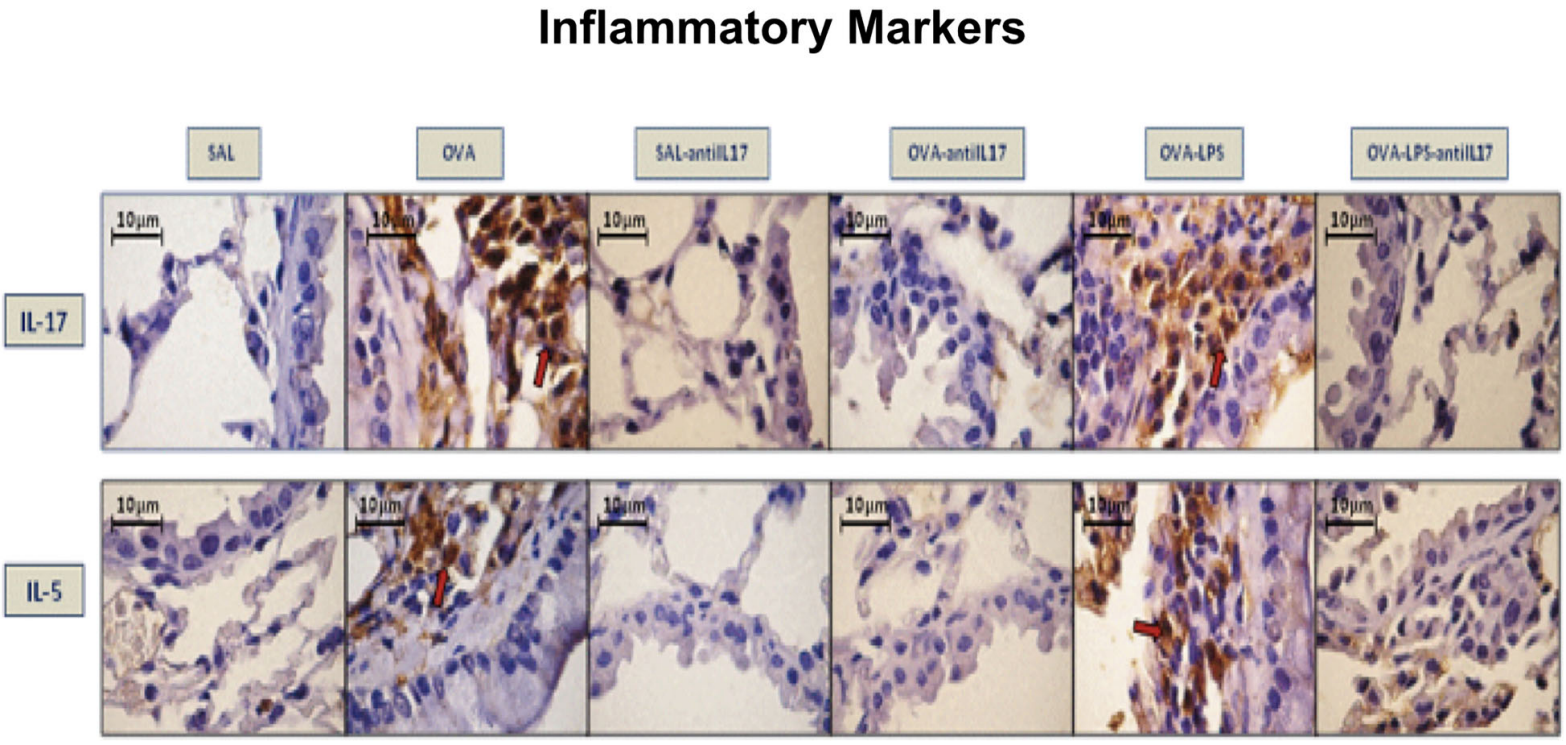
Qualitative analysis of inflammation. Photomicrographs showing airway wall tissue stained to detect inflammatory markers interleukin (IL)-17- and IL-5-expressing cells. SAL: saline; OVA: ovalbumin; LPS: lipopolysaccharide. Scale bars, 10 μm.

**Figure 7:**
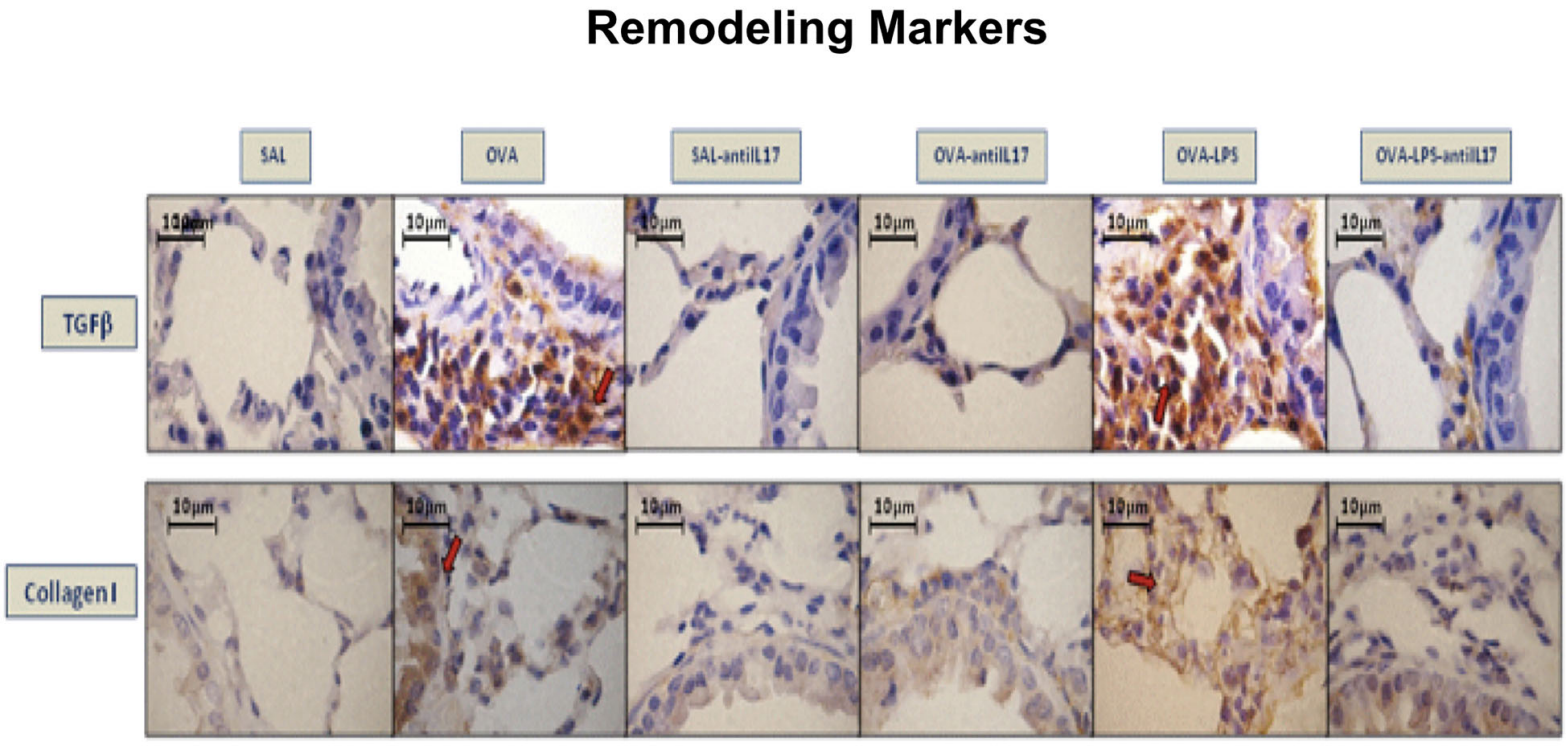
Qualitative analysis of extracellular matrix remodeling. Photomicrographs showing airway wall tissue stained to detect TGF-β and type I collagen expression in the experimental groups. SAL: saline; OVA: ovalbumin; LPS: lipopolysaccharide. Scale bars, 10 μm.

**Figure 8:**
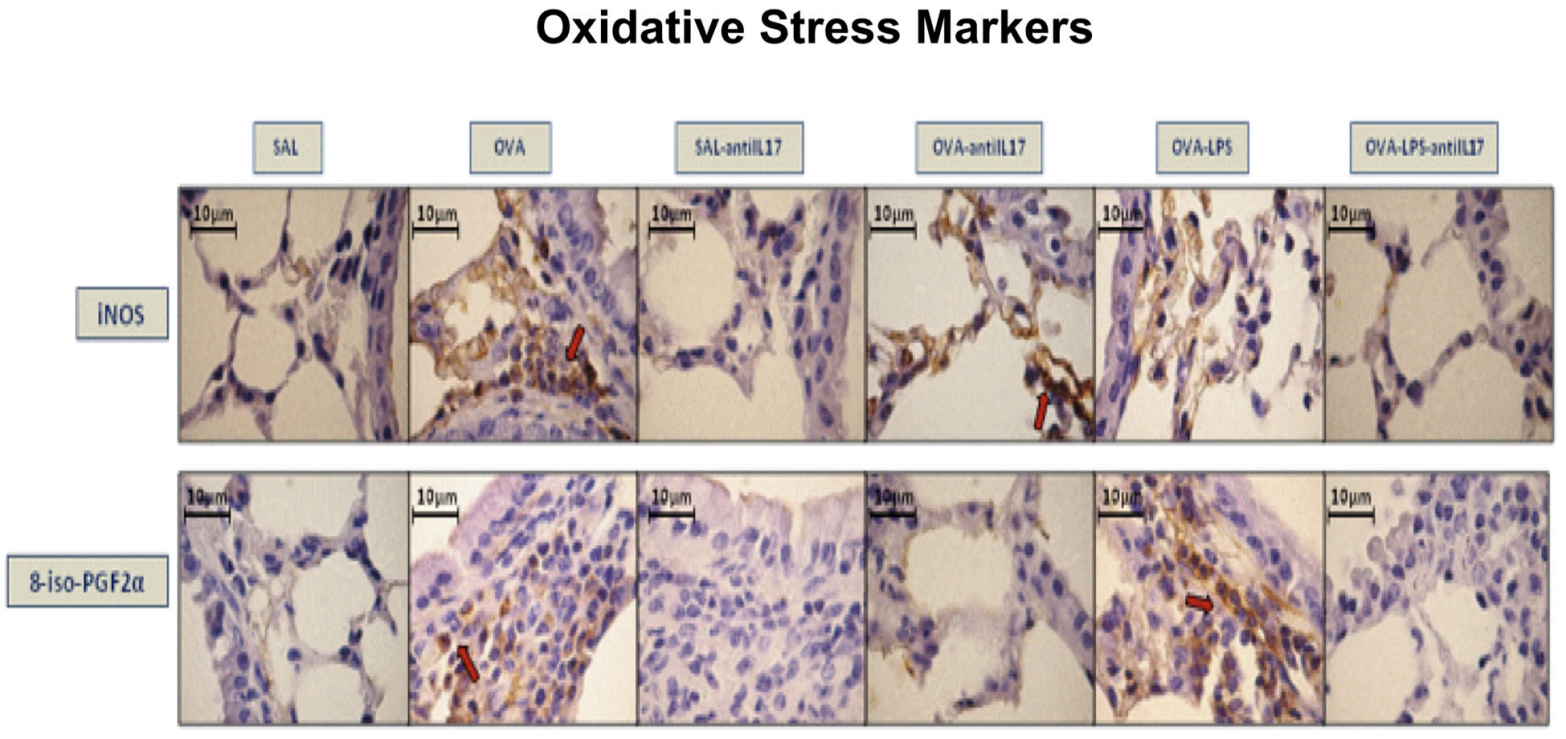
Qualitative analysis of oxidative stress. Photomicrographs showing airway wall tissue stained to detect iNOS- and 8-iso-PGF2α-expressing cells in the experimental groups. iNOS: inducible nitric oxide synthase; SAL: saline; OVA: ovalbumin; LPS: lipopolysaccharide. Scale bars, 10 μm.

Photomicrographs confirmed the presence of inflammation, remodeling, and oxidative stress in the airways based on the expression of IL-17 and IL-5 (Figure 6), TGF-β and type 1 collagen fibers (Figure 7), and iNOS and 8-iso-PGF2α (Figure 8), respectively. Animals exposed only to ovalbumin and ovalbumin plus LPS showed prominent increases in the number of positive cells compared to the control group animals (SAL group). Treatment with anti-IL-17 in the SAL-anti-IL-17, OVA-anti-IL-17, and OVA-LPS-anti-IL-17 groups attenuated the expression of these markers in both groups.

## Discussion

In this study, we demonstrated that IL-17 modulation exerted significant beneficial effects on multiple aspects of pulmonary pathophysiology in an experimental model of chronic airway inflammation, including reduction of bronchial hyperresponsiveness, attenuation of the activation of pro-inflammatory pathways (NF-κB, Rho-kinase), decreased neutrophilic influx, and extracellular matrix remodeling, reinforcing the central role of this cytokine in perpetuating inflammation and lung dysfunction. These results support IL-17 as a potential therapeutic target in neutrophilic inflammatory respiratory diseases that are refractory to conventional therapies. The findings on lung mechanics demonstrated that IL-17 inhibition resulted in improved airway resistance and compliance, evidencing reduced bronchial hyperresponsiveness, a characteristic pathophysiological aspect of diseases such as severe asthma and chronic obstructive pulmonary disease (COPD). These data suggest that airway constriction results from the effects of LPS, which induces NO and isoprostane production, in addition to enhancing smooth muscle responses. This effect was significantly modified by anti-IL-17 treatment. In line with this hypothesis, reduced expression of iNOS, arginase, and ROCK1 attenuated the contractile response of the airways. Notably, significant control of maximal elastance was observed in animals with LPS-induced asthma exacerbation, although with no relevant impact on total respiratory system resistance. On the other hand, Camargo et al. (7) demonstrated that anti-IL-17 administration promoted reduced resistance in both proximal and distal airways in an experimental model of asthma and asthma-COPD overlap, evidencing the therapeutic relevance of immunological modulation in the control of respiratory mechanics.

Dos Santos et al. (13) reported that the administration of an anti-IL-17 antibody combined with a ROCK inhibitor helped modulate several alterations in lung function. Bronchial hyperresponsiveness was attenuated when the antibody was administered together with the treatment, contrary to the results obtained with isolated treatment NF-κB activation, which is reported to exacerbate airway inflammation in asthma. Results from animal studies demonstrated the presence of NF-κB in the lungs after allergen challenge (12). Camargo et al. (4) and dos Santos et al. (13) reported that anti-IL-17 antibodies control and attenuate the responses of NF-κB, ROCK1, and ROCK2 signaling pathways. Therefore, we suggest that the observed effects on bronchial hyperresponsiveness control associated with anti-IL-17 treatment may depend on ROCK expression. Several authors have suggested the importance of oxidative stress responses and NO-arginase pathway activation in the pathophysiology of asthma, revealing a correlation between these responses and asthma symptom severity, airflow limitation, hyperreactivity, and airway remodeling (20).

Levels of eNO and iNOS are associated with IL-17A levels (21). Some studies have demonstrated the role of IL-17A in murine models of allergic asthma and reported that oxidant production in the airways may help regulate IL-17A production and CD4+ T cells in systemic circulation (22). Other studies have shown that oxidative stress activates Th17 responses in the airways (23). To date, only Camargo et al. (16) have explored the role of anti-IL-17 therapy in a model of asthma exacerbation, but without addressing functional and structural airway changes or investigating the involvement of anti-inflammatory cholinergic effects. Our findings advance this knowledge by demonstrating, through PCR analysis, that IL-17 inhibition significantly reduced arginase expression in lung tissue, suggesting a beneficial effect not only on inflammation but also on L-arginine metabolism, associated with bronchial hyperresponsiveness and remodeling. Additionally, recent studies indicate that anti-IL-17 therapy may also exert positive effects on inflammatory profiles overlapping asthma and COPD (7), reinforcing its therapeutic potential in complex and refractory phenotypes.

CCL11 mRNA and protein expression increases at sites of allergic inflammation in asthmatic patients. The increased expression of CCL11 depends on TNF-α and IL-4, which stimulate CCL11 transcription in fibroblasts and pulmonary epithelial cells (24). We hypothesized that the decreased CCL11 expression and eosinophil abundance in groups treated with anti-IL-17 antibody resulted from the suppression of other markers that mediate the CCL11 signaling pathway, such as TNF-α, IL-4, and NF-κB. Our findings are in agreement with Matsukura et al. (25) who showed that TNF-α and IL-4 stimulate CCL11 gene expression by activating NF-κB and STAT6. In a model of allergic inflammation, Th2 cytokines suppressed Th17 responses. Neutralization of IL-4 and/or IL-13 increases Th17 cell infiltration and neutrophilic lung inflammation. However, neutralization of IL-13 and IL-17 protects animals from eosinophilia, mucosal hyperplasia, and airway hyperreactivity, as well as abolishes neutrophilic inflammation, suggesting that combined therapies controlling Th2 and Th17 responses may maximize therapeutic efficacy in asthmatic patients (26), as observed in our results. This confirms that Th2 cells drive eosinophilic inflammation in asthma and that treatments reducing these cytokines can inhibit such inflammation (27). Treatment with an anti-IL-17A antibody attenuated IL-4, IL-5, and IL-13 responses in sensitized animals (OVA + anti-IL-17 and OVA + LPS + anti-IL-17). These results are consistent with previous studies suggesting that anti-IL-17A treatment inhibits Th2-mediated eosinophilic inflammation (14).

Tregs play a vital role in controlling immune responses and exert their effects via anti-inflammatory cytokines such as IL-10 and TGF-β; their deficiency correlates with asthma severity (28). TGF-β, a cytokine with immunomodulatory properties, induces IL-10 secretion by macrophages and Tregs (29). We hypothesize that the elevation in IL-10 expression in our chronic asthma model associated with LPS would be modulated by increased TGF-β levels.

The increase in IL-17-producing cells has been correlated with neutrophil recruitment in an asthma model (30). In a murine model of asthma, IL-17A neutralization significantly reduced neutrophil infiltration (31). Consistent with our results, these data suggest that the Th17 response may contribute to asthma pathogenesis and that its modulation may be an effective control strategy. We hypothesized that anti-IL-17 treatment interferes with the anti-inflammatory cholinergic system. VAChT activity and consequent endogenous ACh release contribute to the anti-inflammatory cholinergic system, and ACh release levels depend on VAChT expression. Recently, it was demonstrated that VAChT is necessary for maintaining pulmonary homeostasis in mice (15). Previous studies have demonstrated that VAChT mRNA expression increases in murine models of chronic allergic airway inflammation, with or without LPS (16-32). Furthermore, anti-IL-17 treatment attenuated VAChT levels, suggesting that VAChT expression is associated with pulmonary inflammation severity. Collagen production, airway hypertrophy and hyperplasia, as well as mucus production increase in murine and guinea pig models of experimental chronic allergic asthma (33). Type III collagen fibers are produced by smooth muscle and reticular cells and cause thickening of the reticular basement membrane; thus, their expression is strongly correlated with hyperresponsiveness. In contrast, type I collagen fibers are produced by fibroblasts and have greater tensile strength (32). These findings suggest that anti-IL-17 treatment may attenuate inflammation and remodeling in an experimental asthma model. The findings of this study corroborate previous reports showing altered collagen fiber density in the airways of ovalbumin-sensitized mice treated with anti-IL-17 (14).

TGF-β stimulates collagen production and is considered a potential remodeling marker in asthma. In severe asthma cases, increased deposition of type I and III collagen and increased smooth muscle mass have been observed in bronchial biopsies (34). The proteoglycans decorin, biglycan, and lumican play crucial roles in the interaction of collagen fibrils with each other and with other components of the extracellular matrix. Biglycan and decorin stabilize collagen fibrogenesis in tissues (35). Therefore, increased decorin expression may be a protective mechanism to modulate pulmonary remodeling (36). However, excessive decorin may regulate and stabilize collagen fiber spacing, creating a stiffer matrix, which can affect the overall elasticity of lung tissues (37). Decorin is also involved in regulating TGF-β function. TGF-β promotes Treg differentiation, which participates in the healing response to inflammation (38). In this study, TGF-β expression was reduced in response to anti-IL-17 treatment. Fibronectin also plays an essential role in morphological regulation during lung development. In asthma, fibronectin and type I and III collagen levels are increased in the smooth muscles of airway walls (39).

The deposition of decorin, biglycan, lumican, and fibronectin was increased in our experimental model. MMP-9 and TIMP-1 expression increased in sensitized animals, but this alteration was reversed by anti-IL-17 treatment. MMP-9 is a marker of airway inflammation and remodeling in patients with severe asthma; it is often detected at high concentrations in plasma and BALF, and its expression is accompanied by a decreased TIMP-1 response in patients with acute exacerbation or severe asthma (40). The number of MMP-9 positive cells increased in the chronic allergic inflammation model, as well as in the asthma model with LPS-induced exacerbation. Our data also indicated increased TIMP-1 expression. We believe that, in addition to inhibiting MMP-9, TIMP-1 has other biological functions. MMP-12 mRNA and protein expression increased in the airways of murine models of allergic inflammation (36). To the best of our knowledge, this is the first study to demonstrate the effects of anti-IL-17 treatment on airway cells and its role in controlling MMP-12, MMP-9, TIMP-1, total collagen, decorin, biglycan, lumican, fibronectin, and TGF-β in a murine model of chronic allergic inflammation with LPS-induced exacerbation.

Finally, we hypothesized that the control of anti-IL-17 responses, hyperresponsiveness, extracellular matrix remodeling, and oxidative stress in the LPS-induced asthma exacerbation model may be related to the expression of NF-κB, ROCK1, and VAChT. We propose that anti-IL-17 treatment may be safe, as it helped improve inflammation, hyperresponsiveness, oxidative stress, and extracellular matrix remodeling in the airways of a murine model of chronic allergic inflammation with LPS-induced exacerbation. IL-17 inhibition helped control bronchial hyperresponsiveness, Th1/Th2/Th17-mediated inflammation, chemokine expression, tissue remodeling, NO-arginase expression, and oxidative stress activation through the modulation of NF-κB, ROCK1, ROCK2, VAChT, and ARG-1 activation in a model of asthma with LPS-induced exacerbation.

Although our findings are relevant, this study presents some limitations that should be considered. First, extrapolation of the results obtained in the experimental model to human pathophysiology requires caution, due to the complexity and heterogeneity of the pulmonary microenvironment in humans. Furthermore, the temporal analysis of the effects of IL-17 inhibition was limited, restricting the assessment of chronic changes in tissue remodeling. Although we demonstrated benefits in pulmonary remodeling, it is not possible to affirm whether these effects are sustained over time.

In conclusion, our study presents a new therapeutic perspective for the treatment of asthma with exacerbation, demonstrating that IL-17 blockade is capable of modulating multiple critical signaling pathways in the disease pathogenesis. We demonstrated that IL-17 inhibition resulted in the control of bronchial hyperresponsiveness, reduction of Th1-, Th2-, and Th17-mediated inflammation, decreased chemokine expression, and attenuation of airway remodeling. Moreover, we observed improvements in oxidative and inflammatory stress parameters, with a consequent positive impact on pulmonary mechanics, highlighting the relevance of the IL-17/NF-κB/VAChT/Rho-kinase pathways as potential therapeutic targets. Thus, our findings reinforced the importance of IL-17 blockade as a promising strategy for the management of severe asthma, especially in exacerbation contexts.

## Data Availability

All data generated or analyzed during this study are included in this published article.

## References

[1] Ji T, Li H (2023). T-helper cells and their cytokines in pathogenesis and treatment of asthma. Front Immunol.

[2] Nightingale S (2020). Role of IL-17 and IL-23 in the pathogenesis of neutrophilic asthma. Int J Immunol Immunother.

[3] Kudo M, Melton AC, Chen C, Engler MB, Huang KE, Ren X (2012). IL-17A produced by αβ T cells drives airway hyper-responsiveness in mice and enhances mouse and human airway smooth muscle contraction. Nat Med.

[4] Camargo LN, dos Santos TM, de Andrade FCP, Fukuzaki S, Lopes FDTQS, Martins MA (2020). Bronchial vascular remodeling is attenuated by anti-IL-17 in asthmatic responses exacerbated by LPS. Front Pharmacol.

[5] Wei Q, Liao J, Jiang M, Liu J, Liang X, Nong G (2021). Relationship between Th17-mediated immunity and airway inflammation in childhood neutrophilic asthma. Allergy Asthma Clin Immunol.

[6] Lukacs NW (2001). Role of chemokines in the pathogenesis of asthma. Nat Rev Immunol.

[7] Camargo LN, Righetti RF, de Almeida FM, dos Santos TM, Fukuzaki S, Martins NAB (2023). Modulating asthma-COPD overlap responses with IL-17 inhibition. Front Immunol.

[8] Prado CM, Martins MA, Tibério IFLC (2011). Nitric oxide in asthma physiopathology. ISRN Allergy.

[9] Li H, Liang Y, Deng J, Cheng Y, Chen S, Lian X (2025). Targeting arginine metabolism in immune cells for the treatment of pulmonary inflammatory diseases. Curr Allergy Asthma Rep.

[10] Ather JL, Hodgkins SR, Janssen-Heininger YMW, Poynter ME (2011). Airway epithelial NF-kB activation promotes allergic sensitization to an innocuous inhaled antigen. Am J Respir Cell Mol Biol.

[11] Aristoteles LRCRB, Righetti RF, Pinheiro NM, Franco RB, Starling CM, da Silva JCP (2013). Modulation of the oscillatory mechanics of lung tissue and the oxidative stress response induced by arginase inhibition in a chronic allergic inflammation model. BMC Pulm Med.

[12] Kume H (2021). Role of airway smooth muscle in inflammation related to asthma and COPD. Adv Exp Med Biol.

[13] dos Santos TM, Righetti RF, Rezende BG, Campos EC, Camargo LN, Saraiva-Romanholo BM (2020). Effect of anti-IL17 and/or Rho-kinase inhibitor treatments on vascular remodeling induced by chronic allergic pulmonary inflammation. Ther Adv Respir Dis.

[14] Gori S, Vermeulen M, Remes-Lenicov F, Jancic C, Scordo W, Ceballos A (2017). Acetylcholine polarizes dendritic cells toward a Th2-promoting profile. Allergy.

[15] Pinheiro NM, Miranda CJCP, Perini A, Câmara NOS, Costa SKP, Alonso-Vale MIC (2015). Pulmonary inflammation is regulated by the levels of the vesicular acetylcholine transporter. PLoS One.

[16] Camargo LN, Righetti RF, Aristóteles LRCRB, dos Santos TM, de Souza FCR, Fukuzaki S (2018). Effects of anti-IL-17 on inflammation, remodeling, and oxidative stress in an experimental model of asthma exacerbated by LPS. Front Immunol.

[B17] Pontes M, Coordenadora MP, Akimovna E, Rivera -Titular B (2019). Ministro De Estado Da Ciência, Tecnologia, Inovações E Comunicações Conselho Nacional De Controle De Experimentação Animal Presidente Conselheiros: I-Representantes do Ministério da Ciência, Tecnologia, Inovações e Comunicações: Renata Mazaro e Costa-Titular Helder Lima de Queiroz-Suplente II-Representantes do Conselho Nacional de Desenvolvimento Científico e Tecnológico.

[B18] Prado CM, Leick-Maldonado EA, Yano L, Leme AS, Capelozzi VL, Martins MA (2006). Effects of nitric oxide synthases in chronic allergic airway inflammation and remodeling. Am J Respir Cell Mol Biol.

[B19] Weibel ER (2010). The challenge of measuring lung structure. On the ‘Standards for the Quantitative Assessment of Lung Structure'. Nihon Kokyuki Gakkai Zasshi.

[B20] Brussino L, Badiu I, Sciascia S, Bugiani M, Heffler E, Guida G (2010). Oxidative stress and airway inflammation after allergen challenge evaluated by exhaled breath condensate analysis. Clin Exp Allergy.

[B21] Chien JW, Lin CY, Yang KD, Lin CH, Kao JK, Tsai YG (2013). Increased IL-17A secreting CD4+ T cells, serum IL-17 levels and exhaled nitric oxide are correlated with childhood asthma severity. Clin Exp Allergy.

[B22] Al-Harbi NO, Nadeem A, Al-Harbi MM, Ansari MA, AlSharari SD, Bahashwan SA (2016). Airway oxidative stress causes vascular and hepatic inflammation via upregulation of IL-17A in a murine model of allergic asthma. Int Immunopharmacol.

[B23] Meurs H, Maarsingh H, Zaagsma J (2003). Arginase and asthma: novel insights into nitric oxide homeostasis and airway hyperresponsiveness. Trends Pharmacol Sci.

[B24] Meyer-Hoffert U, Lezcano-Meza D, Bartels J, Montes-Vizuet AR, Schröder JM, Teran LM (2003). Th2- and to a lesser extent Th1-type cytokines upregulate the production of both CXC (IL-8 and Gro-Alpha) and CC (RANTES, Eotaxin, Eotaxin-2, MCP-3 and MCP-4) chemokines in human airway epithelial cells. Int Arch Allergy Immunol.

[B25] Matsukura S, Stellato C, Plitt JR, Bickel C, Miura K, Georas SN (1999). Activation of eotaxin gene transcription by NF-kappa B and STAT6 in human airway epithelial cells. J Immunol.

[B26] Kim D, McAlees JW, Bischoff LJ, Kaur D, Houshel LK, Gray J (2019). Combined administration of anti-IL-13 and anti-IL-17A at individually sub-therapeutic doses limits asthma-like symptoms in a mouse model of Th2/Th17 high asthma. Clin Exp Allergy.

[B27] Zhang C, Song Y, Wang C, Zhao L, Kang H, Ma X (2017). The effects of chrysophanol on ovalbumin (OVA)-induced chronic lung toxicology by inhibiting Th17 response. Toxicol Mech Methods.

[B28] Willis CR, Siegel L, Leith A, Mohn D, Escobar S, Wannberg S (2015). IL-17RA signaling in airway inflammation and bronchial hyperreactivity in allergic asthma. Am J Respir Cell Mol Biol.

[B29] Rigas D, Lewis G, Aron JL, Wang B, Banie H, Sankaranarayanan I (2017). Type 2 innate lymphoid cell suppression by regulatory T cells attenuates airway hyperreactivity and requires inducible T-cell costimulator-inducible T-cell costimulator ligand interaction. J Allergy Clin Immunol.

[B30] Choy DF, Hart KM, Borthwick LA, Shikotra A, Nagarkar DR, Siddiqui S (2015). TH2 and TH17 inflammatory pathways are reciprocally regulated in asthma. Sci Transl Med.

[B31] Park SJ, Lee YC (2010). Interleukin-17 regulation: an attractive therapeutic approach for asthma. Respir Res.

[32] Prado VF, Roy A, Kolisnyk B, Gros R, Prado MAM (2013). Regulation of cholinergic activity by the vesicular acetylcholine transporter. Biochem J.

[33] Mcmillan SJ, Lloyd CM (2004). Prolonged allergen challenge in mice leads to persistent airway remodelling. Clin Exp Allergy.

[34] Benayoun L, Druilhe A, Dombret MC, Aubier M, Pretolani M (2003). Airway structural alterations selectively associated with severe asthma. Am J Respir Crit Care Med.

[35] Kalamajski S, Oldberg A (2010). The role of small leucine-rich proteoglycans in collagen fibrillogenesis. Matrix Biol.

[36] Pouladi MA, Robbins CS, Swirski FK, Cundall M, McKenzie ANJ, Jordana M (2004). Interleukin-13-dependent expression of matrix metalloproteinase-12 is required for the development of airway eosinophilia in mice. Am J Respir Cell Mol Biol.

[37] Gubbiotti MA, Vallet SD, Ricard-Blum S, Iozzo RV (2016). Decorin interacting network: A comprehensive analysis of decorin-binding partners and their versatile functions. Matrix Biol.

[38] Sanjabi S, Oh SA, Li MO (2017). Regulation of the immune response by TGF-β: From conception to autoimmunity and infection. Cold Spring Harb Perspect Biol.

[39] El-Moneum NA, Mohamed-Hussain AAR, Mohammed EF, Mohamed HO, Mahmoud MMT (2015). Matrix metalloproteinase-9 (MMP-9) and tissue inhibitor of metalloproteinase-1 (TIMP-1) as non-invasive biomarkers of remodelling in asthma. J Pulm Respir Med.

[40] Naik SP, Mahesh PA, Jayaraj BS, Madhunapantula SRV, Jahromi SR, Yadav MK (2017). Evaluation of inflammatory markers interleukin-6 (IL-6) and matrix metalloproteinase-9 (MMP-9) in asthma. J Asthma.

